# Sexual Satisfaction and Associated Biopsychosocial Factors in Stroke Patients Admitted to Specialized Cognitive Rehabilitation

**DOI:** 10.1016/j.esxm.2021.100424

**Published:** 2021-08-30

**Authors:** Jannike K. Vikan, Hildegun Snekkevik, Marie I. Nilsson, Johan K. Stanghelle, Amy Østertun Geirdal, Kerstin S. Fugl-Meyer

**Affiliations:** 1 Department of Neurobiology, Care Science and Society, Karolinska Institutet, Stockholm, Sweden; 2 Sunnaas Rehabilitation Hospital, Nesodden, Norway; 3 Function Area Social Work in Health, Karolinska University Hospital, Stockholm, Sweden; 4 Institute of Clinical Medicine, Medical Faculty, University of Oslo, Oslo, Norway; 5 Faculty of Social Sciences, Department of Social Work, Child Welfare and Social Policy, OsloMet - Oslo Metropolitan University, Oslo, Norway

**Keywords:** Sexual Satisfaction, Sexual complaint, Sexual activity, Sexuality, Stroke rehabilitation, Biopsychosocial

## Abstract

**Introduction:**

The consequences of stroke on sexual life in stroke patients in need of specialized cognitive rehabilitation have been limited explored. A biopsychosocial perspective in post-stroke sexuality studies is warranted to capture the complex picture of stroke consequences and sexual life after stroke and sexual satisfaction is an important outcome measure when exploring such multifactorial associations.

**Aim:**

To explore sexual satisfaction and associated biopsychosocial factors in stroke patients admitted to specialized cognitive rehabilitation.

**Methods:**

A cross-sectional study was performed including 91 consecutive stroke patients admitted to specialized cognitive rehabilitation. Data were collected from medical records and by face-to-face interviews using a structured interview guide and questionnaires. Descriptive and inferential statistics were applied.

**Main outcome measures:**

A wide range of biopsychosocial variables including medical and sociodemographic characteristics, social support, sexual complaints, aspects of sexual life, psychological distress and life satisfaction were analyzed in relation to the main outcome “Satisfaction with sexual life.”

**Results:**

Only 33 % were satisfied with sexual life. Prevalence of sexual complaints was high, more frequent in women (84%) than in men (64%). Three-quarters were less sexually active than before stroke. Multivariable analyses showed that anxiety, sleep problems, manifested sexual complaint, decrease in sexual activity and fear of partner rejection were significantly associated with low odds of sexual satisfaction, while affectionate support and partnership satisfaction were significant for sexual satisfaction. When combined in a biopsychosocial multivariable model only fear of partner rejection (OR 0.07; 95 % CI: 0.01–0.42) and decrease in sexual activity (OR 0.11; 95 % CI: 0.02–0.58) showed significant contribution to sexual satisfaction.

**Conclusion:**

The variety of predictors for sexual satisfaction indicates that therapeutic actions need to be individualized and points towards a broad assessment and interventional approach to meet the sexual rehabilitation needs of stroke patients with cognitive impairments in need of specialized rehabilitation.

**Vikan JK, Snekkevik H, Nilsson MI, et al. Sexual Satisfaction and Associated Biopsychosocial Factors in Stroke Patients Admitted to Specialized Cognitive Rehabilitation. Sex Med 2021;9:100424.**

## INTRODUCTION

Life satisfaction is found to be low after stroke, and sexual life is one of the most vulnerable domains.^1^^,^^2^ A stroke may affect multiple domains such as physical and cognitive function, behavior, as well as psychological and psychosocial aspects of life, causing a wide range of activity limitations and participation restrictions,^3^^,^^4^ which may all affect sexual life.

To our knowledge, previous research on post-stroke sexuality have overlooked patients with poststroke cognitive impairments (PSCI)^5^ in need of cognitive rehabilitation. PSCI, such as problems with attention, concentration, memory, language, visuospatial abilities and executive functions are common poststroke, and frequent in patients with good physical recovery, with extensive impact on functional outcome, activity and participation and quality of life.^5^, ^6^, ^7^, ^8^ Consequently, a large group of stroke patients with long-term disabilities is left with unknown sexual concerns and sexual rehabilitation needs.

In general, sexuality poststroke is poorly addressed in stroke rehabilitation, and several barriers that prevent patients and partners from receiving adequate sexual rehabilitation have been identified.^9^, ^10^, ^11^ The scarcity of a biopsychosocial approach in clinical practice and research investigating sexuality poststroke represents one such barrier. Thus, evidence-based studies to guide clinical practice after stroke from a biopsychosocial perspective are called for to meet the diverse sexual rehabilitation needs,^12^, ^13^, ^14^, ^15^ also in stroke patients with cognitive impairments.

Research on poststroke sexuality have predominantly investigated different aspects of sexual function as main outcome and some focused on sexual activity,^16^ and a majority investigated male sexual dysfunction and patients aged over sixty.^12^^,^ ^14^ Sexual dysfunctions and sexual inactivity are found to be associated with medications prescribed after stroke, coexisting medical conditions, depression and anxiety,^10^^,^^12^^,^^14^^,^^17^ as well as physical impairment and level of independence in activities of daily living.^10^^,^^18^ However, studies show that even individuals with no or mild physical disabilities poststroke experience sexual dysfunctions and sexual dissatisfaction.^19^^,^^20^ Communication disorders are also found to affect sexual life negatively.^21^ How stroke characteristics contribute to sexual dysfunction is inconclusive.^12^^,^^13^ Emotional and relational factors are important contributors to sexual function, sexual activity and satisfaction poststroke,^12^^,^^14^^,^^17^^,^^21^ however; few studies have investigated psychosocial factors in a broader perspective.

Although identifying predictors for sexual dysfunctions is of utmost importance for clinical purposes and sexual well-being, sexual dysfunctions *per se* do not necessarily cause distress or sexual dissatisfaction, and thus cannot explain or determine experiences of sexual well-being alone.^22^ Likewise, experiencing sexual life as dissatisfying does not imply the presence of sexual dysfunction but may be related to other factors.^22^ Thus, focusing mainly on physical sexual function gives an incomplete understanding of sexuality poststroke.^23^

Satisfaction with sexual life is recognized as a key aspect of sexual health and well- being^24^ and was chosen as main outcome in this study. Exploring satisfaction with sexual life allows for revealing the unique subjective experiences of sexual concerns beyond sexual function, acknowledging the diversity of factors possibly influencing sexual satisfaction after stroke. Only 2 studies were identified with sexual satisfaction as the independent variable, concluding that psychosocial factors were important contributors to satisfaction with sexual life.^19^^,^^25^ Thus, little is known about what factors influence satisfaction with sexual life after stroke in a broader perspective, and to our knowledge, no studies have investigated sexual life and satisfaction in a population of stroke patients with verified cognitive impairments applying a biopsychosocial approach.

### Objectives

The aim of the present study was to explore satisfaction with sexual life and identify associations to satisfaction with sexual life in first stroke patients admitted to specialized cognitive rehabilitation. We hypothesized that predictors would be multifactorial and sought to provide a biopsychosocial prediction model for satisfaction with sexual life.

## METHOD

### Participants and Design

Stroke patients admitted to inpatient specialized cognitive rehabilitation at Sunnaas Rehabilitation Hospital (Norway) were consecutively recruited during the period of June 2018–July 2019. Inclusion criteria were adults (> 18 years) with a first stroke. Participants of all gender identities and sexual orientation were welcome to participate. Exclusion criteria were severe medical conditions preventing patients from adequate communication and informed consent. Of 128 eligible patients invited, 28 declined participation, 8 did not respond and one did not receive the invitation due to early discharge. Thus, analyses for the current cross-sectional study included 91 patients (71 % of eligible individuals). The rehabilitation program had a multidisciplinary approach and followed an evidence-based Cognitive Rehabilitation Manual^26^ with local adjustments.^27^ Stroke patients admitted to the rehabilitation program had PSCI^5^ which affected everyday life, for example, work, social- and family life. However, all participants were able to manage self-care and live independently in a home-dwelling environment without assistance from health care in the municipality, although some assistance from family members were common. The cognitive profile of patients varied, however, most participant had two or more impaired cognitive domains. Most common cognitive impairments (based on individualized, recent assessment by psychologist and observation) were memory (81 %), attention (76 %), executive functions (71 %), psychomotor skills (52 %), while fewer had problems with visuospatial abilities (23 %) and language (19 %).

### Data Collection Procedures and Measurements

**Figure 1:**
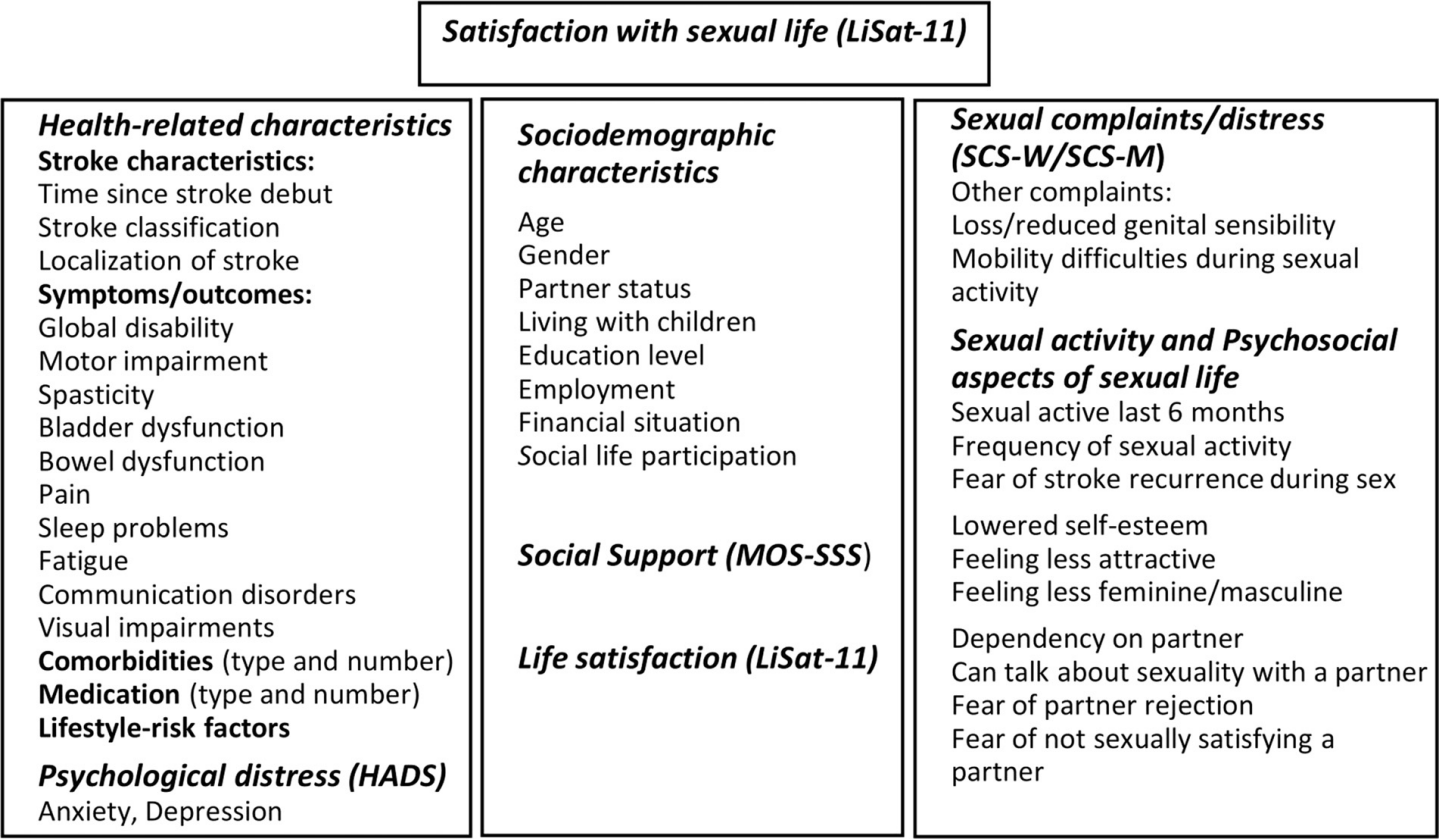
Overview of included variables: Health-related characteristics collected from medical records. Collected by structured face-to-face interview: Psychological distress (HADS: Hospital Anxiety and Depression Scale), Sociodemographic characteristics, Social Support (MOS-SSS, Medical Outcome Study-Social Support Survey), Life Satisfaction including sexual satisfaction (LiSat-11: Life Satisfaction Checklist), Sexual Complaints (SCS-W/SCS-M): Sexual Complaint Screening (female and male version) and Sexual Activity and Psychosocial factors (questions on sexual activity, sexual self-concept and sexual relation).

Information covering a wide range of variables within different biopsychosocial domains (Figure 1) were chosen to explore the complex interaction of independent variables associated to sexual satisfaction. A specialist in stroke rehabilitation (HS) retrieved detailed stroke characteristics and medical variables from patients’ medical records, using a structured coding manual with categorical responses to guide data abstraction and ensure validity and accuracy of data collection.^28^ Additional information was collected from patients using structured face-to-face interviews, conducted by an experienced medical social worker specialized in sexological counselling (JV). A structured study-specific interview guide including sociodemographic factors and psychosocial aspects of sexual life, along with four well-validated questionnaires was used to collect data in the interviews. A research group of experts in stroke rehabilitation and sexual medicine developed the study-specific interview guide. The guide included sections with series of questions within different domains, and all sections had an introduction to the topic and clarification of concepts. The interviews, lasting between 45 and 90 minutes, were conducted during inpatient stays and adapted according to each patient's needs and cognitive function.

#### Sociodemographic Characteristics

The sociodemographic questions included age (continuous), gender (dichotomized into women vs men as none responded “other”), partner relationship (yes vs no), living with children (yes vs no), education level (≤13 vs >13 years), employment status (working/studying full or part-time vs not working/studying), financial situation and social participation prestroke compared to poststroke (dichotomized into unchanged/better vs worsened).

#### Health-related characteristics

Stroke characteristics, symptoms and/or outcomes, comorbidities, medications and life-style risk factors were based on information from the acute hospitalization, stroke imaging, comprehensive clinical examination, observation and assessments made by rehabilitation physicians and the multidisciplinary team during inpatient stay, along with information from patients and significant others.

Level of global disability was classified using the modified Rankin Scale (mRS), with scores ranging from 0 to 6 (0 = no symptoms, 6 = death).^29^ Both functional disabilities and non-physical attributes such as cognition and language were considered when determining level of global disability.^30^ Information concerning cognitive function was based on neuropsychological examination, structured observation by the multidisciplinary team, questionnaires and subjective complaints. Motor impairment was categorized according to level of function (none, mild=limitation in demanding activities, moderate=limitation in activities of daily living, severe=low level of practical function/no function). In statistical analyses, presence of motor impairment is presented as dichotomous (yes vs no). Other medical symptoms or outcomes was dichotomized (yes vs no). Comorbidities are given by most frequent groups of diseases, diseases known to be associated with sexuality and number of comorbidities. Total number of medications used regularly was registered and categorized by drug class. Lifestyle-risk factors (smoking, substance abuse, inactivity and overweight) were dichotomized into yes vs no.

#### Sexual Activity and Psychosocial Aspects of Sexual Life

Frequency of sexual activity was dichotomized into unchanged and/or increased vs decreased compared to levels before stroke. Psychosocial aspects of sexual life covered areas of sexual self-concept and sexual relationship. Responses for ability to talk openly about sexuality with a partner were collapsed into cannot and/or difficult vs yes with ease. All other variables were dichotomized into yes vs no.

#### Questionnaires

The outcome variable “Satisfaction with sexual life” was taken from The “*Life Satisfaction Checklist*” (LiSat-11) which is commonly used to assess life satisfaction poststroke^1^^,^^31^^,^^32^ and found to be valid and reliable.^32^ LiSat-11 include a global item on satisfaction with “life as a whole” and 10 items covering satisfaction within four domains of life: Closeness, Health, Leisure, and Provision. Satisfaction is ranged along a 6-graded ordinal-scale from 1 (very dissatisfied) to 6 (very satisfied). Responses are reported as dichotomous into dissatisfied (score 1–4) and satisfied (5–6) for each item separately.^33^ Lisat-11 showed good internal consistency with a Cronbach's alpha of 0.80.

The “*Medical Outcomes Study-Social Support Survey”* (MOS-SSS), showing good psychometric properties, was used to assess perceived functional social support.^34^ The scale includes four subscales with 19 items altogether (emotional/informational support, tangible support, affectionate support and positive social interaction). Response alternatives are rated on a 5-point Likert scale with higher values indicating higher level of support (1 = never, 2 = rarely, 3 = occasionally, 4 = often, 5 = always). Mean scores are reported for the total scale and subscales (ranging from 1 to 5). The scale showed good internal consistency with a Cronbach's alpha of 0.89–0.96 on subscales and 0.93 for the whole scale.

The “*Sexual Complaint Screener*” (SCS)^35^ with separate version for women (SCS-W) and men (SCS-M) was used for self-reported sexual complaints *per se* after stroke. Response options ranged from 0 (occurring never/almost never) to 4 (occurring almost all the time/always) and were dichotomized into no (score 0–1) and yes (scores 2–4), with “no sexual activity” as an option. “Sexual activity” is defined as any activity performed alone or in an interpersonal context and thus, not limited to sexual intercourse (penetration). Results are also given for complaints occurring often and/or always (manifest). Personal distress related to each of the complaints during the last 6 months was reported ranging from 0 (not at all a problem) to 4 (a very great problem); and dichotomized into no distress (scores 0–1) and yes, distress (scores 2–4). In addition, the SCS includes a question on wishes for follow-up consultation for sexual problems (no *vs* yes). The gender specific scales showed good internal consistency with a Cronbach's alpha of 0.82 for SCS-W and 0.83 for SCS-M.

The “*Hospital Anxiety and Depression Scale*” (HADS) is a commonly used, validated and psychometric sound 14-item self-report screening instrument to assess levels of psychological distress, with one subscale for anxiety [HADS-A] and one for depression [HADS-D].^36^^,^^37^ Every claim has 4 response alternatives ranged from 0 (“not present”) to 3 (“highly present”) providing subscale scores ranging from 0 to 21. A sum score ≥8 on HADS-A and HADS-D indicates possible presence of anxiety and depression.^37^ For HADS-A the Cronbach´s alpha coefficient was 0.83 and for HADS-D 0.79, showing good internal consistency.

### Statistical Analyses

Descriptive data are presented by frequencies, percentages, median and range or mean and standard deviation (SD), with a 95 % confidence interval (CI) for mean differences. The Shapiro-Wilk test was used to examine the normality of distribution. Comparisons between groups were made by Chi-squared test or Fischer's exact for categorical variables and for continuous data student *t-*test for normally distributed data, and Mann-Whitney U test for nonparametric data. Separate analyses for partnered and/or singles were computed were meaningful. Internal consistencies were examined for MOS-SSS, LiSat-11, SCS-W/M and HADS using Cronbach`s alpha. Statistical tests were 2-tailed and significance level was set to *P* ≤ .05 in all analyses.

Independent variables were selected based on prior research, theoretical and clinical relevance. Associations between independent variables and sexual satisfaction were analyzed using univariate and multivariable logistic regression analyses, and the strength of associations expressed as odds ratios (OR) with 95 % confidence intervals (CI). Univariate analyses, presented as crude odds ratios, were computed for all independent variables to examine associations. Separate univariate analyses were computed for women and men to investigate associations between gender-specific sexual complaints and sexual satisfaction. To reduce the risk of losing relevant associations due to low power, variables with *P* value < .25 in univariate logistic regression analyses were included in direct multivariable analyses. Multivariable models were computed to examine the contribution of different groups of domain-specific variables when explaining the variance in sexual satisfaction, presented as adjusted odds ratios. Variables that were rendered significant (*P* < .05) in domain-specific models were entered simultaneously in a final model to identify the combination of independent variables across domains with the strongest prediction of sexual satisfaction. Variables within the same model were tested for multicollinearity and interaction, with the result of excluding MOS-SSS-total from the domain-specific regression model due to collinearity to subscales. Statistical analyses were performed using SPSS version 26.0 (IBM SPSS Inc., Chicago, IL, USA).

### Ethics

This study was approved by the Regional Committee for Medical Research Ethics, East-Norway (REK), (ID ref. 2016/1669). All participants were given oral and written information about the study and signed an informed consent prior to participation.

## RESULTS

### Sociodemographic Characteristics and Perceived Social Support (MOS-SSS)

Of the 91 participants 42 % were women and 58 % men, median age 51 years (range 19–67). A vast majority had a stable partner relationship (76 %). Sociodemographic characteristics and results from MOS-SSS are summarized in Table 1. No significant differences in sexual satisfaction were found for any of the sociodemographic variables, while a significant difference was found for all domains of social support except for tangible support. No significant gender differences (data not shown) were found on any of the sociodemographic variables or social support domains.

**Table 1:** Sociodemographic characteristics and perceived social support (MOS-SSS) in first stroke patients referred to specialized cognitive rehabilitation (n: 91) in relation to satisfaction with sexual life

Variables	Total, n: 91	Satisfied sexual life, n:30	Dissatisfied sexual life, n:61	*P*value
Sociodemographic characteristics				
Age, years, Mean (SD)	48.7 (10.4)	47.2 (10.1)	49.5 (10.5)	.32
*Median (range)*	51 (19-67)	47 (27-64)	51 (19-67)	.25
Gender, n (%)				
Women	38 (41.8)	15 (50.0)	23 (37.7)	.37
Men	53 (58.2)	15 (50.0)	38 (62.3)	
Partner status, n (%)				
Partnered	69 (75.8)	26 (86.7)	43 (70.5)	.15
Single	22 (24.2)	4 (13.3)	18 (29.5)	
Living with children <18 y, n (%)				
Yes	46 (50.5)	17 (56.7)	29 (47.5)	.55
No	45 (49.5)	13 (43.3)	32 (52.5)	
Education level, n (%)				
≤13 y	29 (31.9)	6 (20.0)	23 (37.7)	.14
>13 y	62 (68.1)	24 (80.0)	38 (62.3)	
Work/study*, n = 89†, n (%)				
Yes	31 (34.8)	8 (27.6)	23 (38.3)	.45
No	58 (65.2)	21 (72.4)	37 (61.7)	
Financial situation, n (%)				
Unchanged/better	37 (40.7)	12 (40.0)	25 (41.0)	1.
Worsened	54 (59.3)	18 (60.0)	36 (59.0)	
Social life participation, n (%)				
Unchanged/better	15 (16.5)	4 (13.3)	11 (18.0)	.76
Declined	76 (83.5)	26 (86.7)	50 (82.0)	
MOS-SSS‡*, Mean* (SD)				
Social support, total score	4.2 (0.8)	4.6 (0.4)	4.0 (0.9)	**.002**
Emotional/informational support	4.1 (0.9)	4.4 (0.7)	4.0 (0.9)	**.025**
Tangible support	4.3 (1.0)	4.6 (0.6)	4.2 (1.1)	.28
Affectionate support	4.6 (0.8)	4.9 (0.2)	4.4 (0.9)	**<.001**
Positive social interaction	4.3 (0.9)	4.7 (0.6)	4.1 (0.9)	**.002**

Bold figures indicate significance.

⁎ Full or part-time.

† Retired participants, n = 2, not included in analyses.

‡ Medical Outcome Study – Social Support Survey (MOS-SSS).

### Health-Related Characteristics

Median time since stroke was 24 months (range 3–170) and Ischemic stroke was most frequent (68 %). Table 2 presents Health-related characteristics, of which significant differences in sexual satisfaction were found for sleep problems and anxiety (HADS-A). Gender differences (not shown) were found for type of stroke where women more often had had a subarachnoid hemorrhage than men (32 % vs 11 %, *P* = .008), and men had significantly more medications than women did (4 [range 0–15] vs 3 [range 0–11]; *P* = .025).

**Table 2:** Health-related characteristics in first stroke patients referred to specialized cognitive rehabilitation (n: 91) in relation to satisfaction with sexual life

Health-related characteristics	Total, n:91	Satisfied sexual life, n:30	Dissatisfied sexual life, n:61	*P* value
Time since stroke (mo) *median (range)*	24.0 (3-170)	22.5 (3-170)	24.0 (4-130)	.99
Classification of stroke, n (%)				
Ischemic	62 (68.1)	19 (63.3)	43 (70.5)	.52
Hemorrhage	11 (12.1)	3 (10.0)	8 (13.1)	
Subarachnoid hemorrhage	18 (19.8)	8 (26.7)	10 (16.4)	
Location of stroke (n = 89), n (%)				
Right hemisphere	35 (39.3)	15 (50.0)	20 (33.9)	.33
Left hemisphere	35 (39.3)	8 (26.7)	27 (45.8)	
Bilateral hemisphere	10 (11.2)	4 (13.3)	6 (10.2)	
Cerebellum	9 (10.1)	3 (10.0)	6 (10.2)	
Global disability (mRS*), n (%)				
Slightly (score 2)	82 (90.1)	27 (90.0)	55 (90.2)	1.
Moderate (score 3)	9 (9.9)	3 (10.0)	6 (9.8)	
Motor impairment, n (%)	37 (40.7)	10 (33.3)	27 (44.3)	.44
None	54 (59.3)	20 (66.7)	34 (55.7)	.06
Mild	21 (23.1)	8 (26.7)	13 (21.3)	
Moderate	11 (12.1)	0 (0)	11 (18.0)	
Severe	5 (5.5)	2 (6.7)	3 (4.9)	
Spasticity, n (%)	15 (16.5)	4 (13.3)	11 (18.0)	.77
Bladder dysfunction, n (%)	22 (24.2)	7 (23.3)	15 (24.6)	1.
Bowel dysfunction, n (%)	6 (6.6)	2 (6.7)	4 (6.6)	1.
Pain VAS scale 0-10 (score >3 = moderate/severe), n (%)	34 (37.4)	12 (40.0)	22 (36.1)	.89
Sleep problem, n (%)	40 (44.0)	8 (26.7)	32 (52.5)	**.035**
Fatigue, n (%)	81 (89.0)	28 (93.3)	53 (86.9)	.49
Communication disorders†, n (%)	20 (22.0)	8 (26.7)	12 (19.7)	.63
Visual impairments, n (%)	14 (15.4)	5 (16.7)	9 (14.8)	1.
Comorbidities, n (%)	74 (81.3)	25 (83.3)	49 (80.3)	.95
Number of comorbidities, *median (range)*	2.0 (0-10)	1.0 (0-4)	2.0 (0-10)	.07
Hypertension	20 (22.0)	3 (10.0)	17 (27.9)	.09
Musculoskeletal	17 (18.7)	5 (16.7)	12 (19.7)	.95
Heart diseases	9 (9.9)	1 (3.3)	8 (13.1)	.26
Other brain diseases	9 (9.9)	2 (6.7)	7 (11.5)	.71
Epilepsy	8 (8.8)	2 (6.7)	6 (9.8)	1.
Diabetes	4 (4.4)	0	4 (6.6)	.30
Other somatic diagnoses‡	39 (42.9)	11 (36.7)	28 (45.9)	.54
Psychiatric diagnoses§	10 (11.0)	4 (13.3)	6 (9.8)	.72
Medication, n (%)	80 (87.9)	27 (90.0)	53 (86.9)	1.
Number of medications, *median (range)*	3.0 (0-15)	3.0 (0-11)	4.0 (0-15)	.17
Antithrombotic agents/anticoagulants	58 (63.7)	18 (60.0)	40 (65.6)	.77
Statins	43 (47.3)	12 (40.0)	31 (50.8)	.45
Antihypertensive	32 (35.2)	7 (23.3)	25 (41.0)	.15
Antidepressants	20 (22.0)	5 (16.7)	15 (24.6)	.55
Antiepileptic drugs	18 (19.8)	5 (16.7)	13 (21.3)	.81
PDE-5 inhibitors (n = 58)	6 (10.3)	0	6 (9.8)	.17
Other medicationsǁ	58 (63.7)	16 (53.3)	36 (59.0)	.77
Lifestyle risk factors¶, n (%)	29 (31.9)	8 (26.7)	21 (34.4)	.61
HADS-A♯, sum score ≥8, n (%)	31 (34.1)	5 (16.7)	26 (42.6)	**.026**
HADS-D♯, sum score ≥8, n (%)	23 (25.3)	4 (13.3)	19 (31.1)	.11

Bold figures indicate significance.

⁎ mRS=modified Rankin Scale.

† Primarily expressive communication disorders (word finding difficulties).

‡ Migraine, asthma, hypothyreosis and diagnoses with ≤4 patients affected.

§ ADHD+bipolar/personality disorder/PTSD (n:4), Bipolar (n:2), PTSD (n:3), Personality disorder (n:1).

ǁ Pain killers, sleep medications, proton pump inhibitors, antiallergenic and medications used by ≤4 patients.

¶ Overweight (n:13), smoking (n:12) and substance use (n:5).

♯ HADS=Hospital Anxiety and Depression Scale, HADS-A: subscale anxiety, HADS-D: subscale depression.

### Sexual Complaints Screener (SCS-W/SCS-M)

The most common distressing complaint for women was difficulties reaching orgasm (53 %) and for men lack of and/or reduced sexual interest and/or desire (38 %). Sexual complaints, *per se* as well as distressing, are presented in Table 3. Women more often reported sexual complaints *per se* after stroke (border significant: *P* = .051), and had significantly more complaints than men (3 [range 0–6] vs 1 [range 0–5]; *P* .021). No gender difference for number of distressing complaints was found.

**Table 3:** Prevalence of sexual complaints and distressing sexual complaints (SCS) in female and male first stroke patients referred to specialized cognitive rehabilitation in relation to satisfaction with sexual life

Sexual complaints	Total, n(%)	Satisfied sexual life, n (%)	Dissatisfied sexual life, n (%)	*P*value
Female sexual complaints (SCS-W)	**n:38**	**n:15**	**n:23**	
Lack of/reduced sexual interest/desire	24 (63.2)	6 (40.0)	18 (78.3)	**.04**
*Distressing*	*18 (47.4)*	*4 (26.7)*	*14 (60.9)*	**.05**
Lack of physical sexual excitement (Lubrication)	17 (44.7)	6 (40.0)	11 (52.4)	.49
*Distressing*	*16 (42.1)*	*5 (33.3)*	*11 (47.8)*	.58
Lack of pleasurable sexual feelings	18 (47.4)	5 (33.3)	13 (61.9)	.18
*Distressing*	*16 (42.1)*	*3 (20.0)*	*13 (56.5)*	**.05**
Difficulties reaching orgasm	25 (65.8)	9 (60.0)	16 (76.2)	.38
*Distressing*	*20 (52.6)*	*6 (40.0)*	*14 (60.9)*	.35
Difficulties allowing vaginal penetration	1 (2.6)	na	na	na
Genital pain related to sexual activity	3 (7.9)	0 (0)	3 (13.0)	.20
Persistent and unwanted genital arousal	0	na	na	na
One or more sexual complaints	32 (84.2)	11 (73.3)	21 (91.3)	.30
One or more distressing sexual complaint	*25 (65.8)*	8 (53.3)	17 (73.9)	.34
Sexual complaint experienced often/always	23 (60.5)	5 (33.3)	18 (78.3)	**.015**
Male Sexual complaints (SCS-M)	**n:53**	**n:15**	**n:38**	
Lack of or reduced sexual interest/desire	25 (47.2)	5 (33.3)	20 (52.6)	.33
*Distressing*	*20 (37.7)*	*4 (26.7)*	*16 (42.1)*	.47
Need more stimulation to achieve erection (n:52)	13 (24.5)	1 (6.7)	12 (32.4)	.078
*Distressing*	*12 (22.6)*	*1 (6.7)*	*11 (29.7)*	.078
Difficulties getting/maintain erection	17 (32.1)	3 (20.0)	14 (37.8)	.33
*Distressing*	*15 (28.3)*	*3 (20.0)*	*12 (32.4)*	.50
Premature ejaculation (n:51)	13 (24.5)	1 (7.7)	12 (32.4)	.82
*Distressing*	*10 (18.9)*	*0 (0)*	*10 (27.0)*	**.045**
Difficulty ejaculating/reaching orgasm	12 (22.6)	2 (13.3)	10 (29.3)	.47
*Distressing*	*10 (18.9)*	*1 (6.7)*	*9 (23.7)*	.25
Concerned about size/shape of penis	4 (7.5)	1 (6.7)	3 (7.9)	1.
Genital pain during/shortly after sexual activity	1 (1.9)	na	na	na
One or more sexual complaints	34 (64.2)	7 (46.7)	27 (71.1)	.18
One or more distressing sexual complaint	*30 (56.6)*	6 (40.0)	24 (63.2)	.22
Sexual complaint experienced often/always	21 (39.2)	2 (13.3)	19 (50.0)	**.032**

Bold figures indicate significance.

Those satisfied with sexual life had significantly lower number of sexual complaints *per se* than those dissatisfied (1 [range 0–4] vs 3 [range 0–6]; *P* = .014) and lower number of distressing complaints (0 [range 0–4] vs 3 [range 0–6]; *P* = .007). Patients experiencing manifest sexual complaints (48 %) were more often dissatisfied with sexual life than those with sexual complaints less often (84 % vs 16 %, *P* = .002). However, 32 % of women and 21 % of men reported being satisfied with sexual life in spite of sexual complaints.

Additionally, and with no significant differences related to gender or sexual satisfaction, the stroke-related complaint “difficulties positioning one's body during sexual activity” was reported by 41 %, and “decrease or loss of genital sensibility” by 17 %. A majority (60 %) expressed a need for follow-up consultation for sexual concerns.

### Sexual Activity and Psychosocial Aspects of Sexual Life

A decrease in sexual activity was reported by 75% and 35% had ceased being sexually active. Significant differences in sexual satisfaction were found for all variables within the sexual activity and psychosocial domains except for fear of stroke during sex and feeling dependent on a partner (Table 4). Single participants more often feared being rejected from a partner sexually than those in a partner relationship (64 % vs 35 %; *P* = .032), no other differences were found regarding partner status. Only one of the 10 investigated variables showed significant gender differences (data not shown); that is, women reported lower self-esteem related to sexual life after stroke then men (87 % vs 62 %; *P* = .019).

**Table 4:** Sexual activity and psychosocial aspects of sexual life in first stroke patients referred to specialized cognitive rehabilitation (n: 91) in relation to satisfaction with sexual life

	All, n:91	Satisfied sexual life, n:30	Dissatisfied sexual life, n:61	
Variables	n (%)	n (%)	n (%)	*P*value
Sexually active last 6 mo				
Yes	59 (64.8)	26 (86.7)	33 (54.1)	**.005**
No	32 (35.2)	4 (13.3)	28 (45.9)	
Frequency of Sexual activity				
Unchanged/increased	23 (25.3)	16 (53.3)	7 (11.5)	**<.001**
Decreased	68 (74.7)	14 (46.7)	54 (88.5)	
Fear of stroke recurrence during sex				
Yes	22 (24.2)	6 (20.0)	16 (26.2)	.69
No	69 (75.8)	24 (80.0)	45 (73.8)	
Lower self-esteem				
Yes	66 (72.5)	17 (56.7)	49 (80.3)	**.033**
No	25 (27.5)	13 (43.3)	12 (19.7)	
Feel less attractive				
Yes	53 (58.2)	9 (30.0)	44 (72.1)	**<.001**
No	38 (41.8)	21 (70.0)	17 (27.9)	
Feel less feminine/masculine				
Yes	40 (44.0)	7 (23.3)	33 (54.1)	**.011**
No	51 (56.0)	23 (76.7)	28 (45.9)	
Feel more dependent on a partner				
Yes	55 (60.4)	19 (63.3)	36 (59.0)	.87
No	36 (39.6)	11 (36.7)	25 (41.0)	
Can talk about sexuality with a partner				
Yes, with ease	64 (70.3)	26 (86.7)	38 (62.3)	**.032**
No/difficult	27 (29.7)	4 (13.3)	23 (37.7)	
Fear of partner rejection				
Yes	38 (41.8)	4 (13.3)	34 (55.7)	**<.001**
No	53 (58.2)	26 (86.7)	27 (44.3)	
Fear of not satisfying a partner sexually				
Yes	32 (35.2)	4 (13.3)	28 (45.9)	**.005**
No	59 (64.8)	26 (86.7)	33 (54.1)	

Bold figures indicate significance.

### Life Satisfaction (LiSat-11)

Sexual life was found to be satisfactory in 33%. Significant differences in satisfaction with sexual life were found for life as a whole, partner relationship and leisure time (Table 5). There was no significant difference in satisfaction with partner relationship between partnered and single patients and no gender differences were found for any of the life satisfaction items (data not shown).

**Table 5:** Life satisfaction (LiSat-11) reported by first stroke patients referred to specialized cognitive rehabilitation (n: 91) in relation to satisfaction with sexual life.

	Total, n:91	Satisfied sexual life, n:30	Dissatisfied sexual life, n:61	
Satisfaction with:	n (%)	n (%)	n (%)	*P* value
Life as a whole	20 (22.0)	12 (40.0)	8 (13.1)	**.008**
*Closeness*				
Family life	53 (58.2)	19 (63.6)	34 (55.7)	.64
Partner relationship	56 (61.5)	26 (86.7)	30 (49.2)	**.001**
Sexual life*	30 (33.0)	-	-	-
*Health*				
ADL	73 (80.2)	28 (93.3)	45 (73.2)	.055
Somatic health	20 (22.0)	8 (26.7)	12 (19.7)	.62
Psychological health	29 (31.9)	7 (23.3)	22 (36.1)	.32
*Spare time*				
Leisure	22 (24.2)	12 (40.0)	10 (16.4)	**.027**
Contacts	26 (28.6)	10 (33.3)	16 (26.2)	.65
*Provision*				
Vocation	15 (16.5)	3 (10.0)	12 (19.7)	.39
Economy	32 (35.2)	12 (40.0)	20 (32.6)	.66

Bold figures indicate significance.

⁎ Main outcome measure.

### Associations Between Independent Variables and Sexual Satisfaction

Multivariable models of domain-specific combinations of independent variables each explained between 25% and 32% of the variance in sexual satisfaction (Table 6). Affectional support (OR 5.5; 95% CI: 1.01–30.1) and satisfaction with partner relationship (OR 4.9; 95% CI: 1.4–16.8) were associated with sexual satisfaction, while sleep problems (OR 0.27; 95 % CI: 0.09–0.80), anxiety (OR 0.26; 95% CI: 0.08–0.83), manifest sexual complaint (OR 0.2; 95% CI: 0.05–0.74), decrease in sexual activity (OR 0.16; 95% CI: 0.05–0.50), feeling less attractive (OR 0.24; 95% CI: 0.08–0.79) and fear of partner rejection (OR 0.22; 95% CI: 0.06–0.90) all were associated with low odds of sexual satisfaction.

**Table 6:** Crude and multivariable logistic regression for the association between independent variables and sexual satisfaction in first stroke patients referred to specialized cognitive rehabilitation (n: 91).

Predictor variables	Crude analyses OR (95 % CI)	*P* value	Model 1-8 Multivariable OR (95 % CI)	*P* value	Summary domain-specific models
Sociodemographic characteristics					Model 1
Gender (ref: Women) Men	0.61 (0.25-1.5)	.27	1.7 (0.67-448)	.26	R^2^=0.11
Partnered (ref: No) Yes	2.7 (0.83-8.9)	.10	3.1 (0.91-10.5)	.07	68 % Correct classification
Education level (ref:≤ 13 years) > 13 years	2.4 (0.88-6.8)	.09	2.3 (0.78-6.6)	.13	
Social Support Survey (MOS-SSS)					Model 2
Overall social support	**3.9 (1.6-9.5)**	**.003**	NA		R^2^=0.26
Emotional/ informational support	**1.8 (1.01-3.4)**	**.046**	0.92 (0.43-2.0)	.84	69 % Correct classification
Affectionate support	**8.7 (1.7-44.8)**	**.010**	**5.5 (1.01-30.1)**	**.049**	
Positive social relations	**2.6 (1.345.2)**	**.005**	1.8 (0.78-4.0)	.17	
Health-related characteristics					Model 3
Number of Comorbidities	**0.71 (0.52-0.98)**	**.037**	0.76 (0.52-1.1)	.17	R^2^=0.28
Hypertension (ref: No) Yes	0.29 (0.08-1.1)	.06	0.24 (0.04-1.5)	.12	73 % Correct classification
Sleep problems (ref: No) Yes	**0.33 (0.13-0.85)**	**.022**	**0.27 (0.09-0.80)**	**.018**	
Number of medications	0.89 (0.76-1.1)	.22	1.1 (0.89-1.4)	.33	
Antihypertensive (ref: No) Yes	0.44 (0.16-1.2)	.10	0.83 (0.19-3.6)	.80	
Anxiety (HADS-A) (ref: score <8) score ≥8	**0.27 (0.09-0.80)**	**.018**	**0.26 (0.08-0.83)**	**.023**	
Sexual complaints (SCS-W/SCS-M)					Model 4
Number of distressing sexual complaints	**0.70 (0.53-0.93)**	**.015**	0.67 (0.38-1.19)	.17	R^2^=0.25
Distressing sexual complaints (ref: No) Yes	0.43 (0.17-.1.04)	.062	2.2 (0.5-10.7)	.37	74 % Correct classification
Sexual complaints (≤ occasionally) Often/always	**0.20 (0.07-0.53)**	**.001**	**0.2 (0.05-0.74)**	**.016**	
Sexual activity					Model 5
Sexual activity (ref: Unchanged/increased) Decreased	**0.11 (0.04-0.33)**	**<.001**	**0.16 (0.05-0.50)**	**.02**	R^2^=0.31
Sexually active last 6 months (ref: No) Yes	**5.5 (1.7-17.7)**	**.004**	3.2 (0.90-11.58)	.07	77 % Correct classification
Self-concept					Model 6
Feeling less attractive (ref: No)Yes	**0.17 (0.06-0.43)**	**<.001**	0.24 (0.08-0.79)	**.018**	R^2^=0.28
Feeling less feminine/ masculine (ref: No) Yes	**0.26 (0.10-0.69)**	**.007**	0.56 (0.17-1.83)	.22	75 % Correct classification
Feelings of lower self-esteem (ref: No) Yes	**0.32 (0.12-0.84)**	**.020**	0.43 (0.13-1.4)	.16	
Sexual relation confidence					Model 7
Can talk about sexuality with a partner (ref: No) Yes	**3.9 (1.2-12.7)**	**.022**	2.3 (0.53-9.9)	.26	R^2^=0.32
Fear of partner rejection (ref: No) Yes	**0.12 (0.04-0.39)**	**<.001**	**0.22 (0.06-0.90)**	**.036**	74 % Correct classification
Fear of not satisfying a partner sexually (ref: No) Yes	**0.18 (0.06-0.58)**	**.004**	0.36 (0.10-1.4)	.14	
Life satisfaction					Model 8
Satisfied with Life as a whole (ref: No) Yes	**4.4 (1.6-12.5)**	**.005**	2.0 (0.60-6.7)	.26	R^2^=0.30
Satisfied with Partner relation (ref: No) Yes	**6.7 (2.1-21.6)**	**.001**	**4.9 (1.4-16.8)**	**.011**	73 % Correct classification
Satisfied with independence in ADL (ref: No) Yes	**5.0 (1.1-23.3)**	**.042**	2.9 (0.55-15.3)	.21	
Satisfied with Leisure time (ref: No) Yes	**3.4 (1.3-9.2)**	**.016**	1.9 (0.61-6.1)	.27	

Bold numbers indicate statistical significant values (*P* ≤ .05). Crude analyses: Independent variables reaching significance level *P* < .25 for the association with sexual satisfaction, in addition Gender *P* > .25 included. Model 1-8: Gender and all variables reaching significance level *P* < .25 in crude analyses within same domain entered simultaneously in domain specific models. NA not included in analyses due to collinearity with variables within domain. R^2^ = Nagelkerke, OR= odds ratio; CI= Confidence Interval.

Univariate analyses were conducted for women and men separately; examining associations between gender-specific sexual complaints and sexual satisfaction. In women, only distress related to loss of desire (OR 0.23; 95% CI: 0.06-0.97) and lack of pleasure (OR 0.19; 95% CI: 0.04-0.87) were significantly associated with low odds of sexual satisfaction. In men, a perfect prediction was found for distressing premature ejaculation and sexual dissatisfaction, the model did not converge and no results are reported.

### Biopsychosocial Prediction Model for Sexual Satisfaction

In the final multivariable logistic regression model combining all biopsychosocial domains (Table 7) two variables remained significant: Reduced frequency of sexual activity (OR 0.11; 95% CI: 0.02–0.58) and fear of being rejected by a partner (OR 0.07; 95% CI: 0.01–0.42) were associated with low odds of sexual satisfaction. This combination of independent variables explained 62% of the variance in sexual satisfaction (R^2^ = 0.62), and significantly predicts sexual satisfaction.

**Table 7:** Final multivariable logistic regression model for prediction of sexual satisfaction in first stroke patients referred to specialized cognitive rehabilitation (n: 91)

Predictor variables	Final model 9 Multivariabel OR (CI 95 %)	*P* value	Summary multidomain model
Affectionate support	5.2 (0.75-35.4)	.09	R^2^ = 0.62
Sleep problems (ref: No) Yes	0.33 (0.09-1.3)	.12	84 % Correct classification
HADS-A case (ref: No)Yes	1.1 (0.24-4.9)	.90	
Sexual complaints (≤ occasionally) Often/always	0.44 (0.08-2.3)	.34	
Sexual activity (ref: Decrease) Unchanged/Increased	**0.11 (0.02-0.58)**	**.010**	
Feeling less attractive (ref: No)Yes	0.53 (0.14-2.0)	.36	
Fear of partner rejection (ref: No) Yes	**0.07 (0.01-0.42)**	**.004**	
Satisfied with Partner relation (ref: No) Yes	0.90 (0.16-4.5)	.86	

Bold numbers indicate statistical significant values (*P* <.05). R^2^ = Nagelkerke, OR = Odds Ratio, CI = Confidence Interval

Model 9 = multi-domain model with gender and all significant variables from domain-specific models 1-8 entered simultaneously.

## DISCUSSION

The present study is, to our knowledge, one of the first investigations addressing sexuality in a biopsychosocial context in stroke patients admitted to specialized cognitive rehabilitation. In the current cohort, level of sexual satisfaction was particular low and prevalence of sexual complaints was high compared to previous studies of stroke patients where cognitive dysfunctions were not reported. Whether the lack of reporting on cognitive dysfunctions in previous poststroke sexuality studies can be ascribed to under-recognition or lack of measurement of such impairments^8^ or exclusion from studies is unknown. Our hypothesis that predictors would be multifactorial was confirmed by a large and wide range of variables significantly associated with sexual satisfaction. Psychosocial factors contributed the most to explain the variance and a combination of fear of partner rejection and decrease in sexual activity appears to contribute particularly to dissatisfaction with sexual life. Furthermore, very few gender differences were found across all the different variables investigated.

The prevalence of sexual satisfaction poststroke was among the lowest reported after stroke,^12^^,^^31^ but similar to a study by Roding et al^2^ with comparable age groups, time since stroke onset and stroke severity; as well as a multi-center study of severe stroke in specialized rehabilitation.^1^ Despite patients being young compared to most earlier samples on poststroke sexuality,^12^ a vast majority had fallen out of work and social life. Regardless of huge negative changes and low satisfaction within these areas, none of the sociodemographic characteristics were associated with sexual satisfaction. A positive finding, similar to results by Fugl-Meyer et al,^31^ was the high satisfaction with family life and partner relationship, and not surprisingly, the latter was significantly associated with sexual satisfaction. Results from the present study suggest that being satisfied is more important than the partner-status itself. Thus, sexual satisfaction should be addressed in clinical practice and research, regardless of partner-status.^21^

Social support is well known for contributing to physical and psychological health and well-being, and recognized as important for rehabilitation outcome.^38^, ^39^, ^40^ The overall perceived social support was relatively good in this study sample; however, those dissatisfied with sexual life perceived significantly lower social support within all dimensions of social support. Affectionate support was the only dimension contributing to sexual satisfaction in multivariable analyses, indicating that having someone showing love and affection is particularly important for overall sexual satisfaction. Thus, this is an argument for social support being essential in poststroke sexual rehabilitation.

Somewhat surprising, of the many health-related variables included in the present study only anxiety (HADS-A) and sleep problems contributed to explain the variation in satisfaction with sexual life in the Health-domain model, and none of them did in final multivariable model. Patients frequently reported fears related to confidence in sexual relationships and some feared stroke recurrence during sexual activity. These aspects can possibly be related to anxiety symptoms as a response to current stress, impending threat or fears about the future. Moreover, sleep problems are common after stroke and more pronounced in patients with comorbid depression and anxiety.^41^ The association between sleep problems and sexual dissatisfaction may be explained by these interacting factors. Even though depression is known to be related to sexual dysfunction, no association to sexual satisfaction was found in the present study.

The few associations to health-related characteristics may suggest that even if some of these variables can affect sexual function,^10^^,^^12^^,^^14^^,^^17^ this is not necessarily true for satisfaction with sexual life.

The contribution of emotional and relational factors in the present study strengthens the suggestions that psychological and psychosocial factors play a crucial role in understanding sexuality and sexual problems poststroke, also in patients with good clinical recovery.^10^^,^^42^ Fear of partner rejection seems particular important for sexual satisfaction. Even single patients feared rejection, and as stated by McGrath et.al.^21^ they might be especially vulnerable, where negative thoughts about self and changes to roles and identity may act as barriers to engage in a new intimate relationship. These aspects can often be reinforced by social norms and expectations related to attractiveness and gender roles affecting stroke survivors’ relational confidence and sexuality,^21^^,^^23^^,^^43^ most likely contributing to a vicious cycle of negative self-evaluation and fears.

A decline or absence of sexual activity is quite common after stroke. Barriers for resuming sexual activity are often related to emotional and relational factors, such as partner aspects, difficulties in communication on sexuality, negative changes in self-perception, performance anxiety and fear of having a recurrent stroke and sexual dysfunctions.^10^^,^^18^^,^^25^^,^^42^ In the present study, decreased frequency of sexual activity (not exclusively penetration) was associated with sexual dissatisfaction. Sexual activity is associated with sexual well-being,^10^^,^^12^ and gap between desired and actual frequency may cause dissatisfaction with sexual life as well as within a relationship.^22^

Prevalence of sexual complaints in the present study was among the highest reported compared to previous findings on sexuality after stroke.^12^, ^13^, ^14^ Experiencing manifest sexual complaints is more likely to cause ongoing distress than those occurring occasionally,^44^ explaining the strong association to sexual dissatisfaction. In gender-specific analyses, only sexual complaints perceived as distressing turned out to be significant for sexual satisfactions in both women and men. Assessment of distress related to sexual complaints is highly relevant to determine the clinical relevance of complaints and from a diagnostic point of view crucial,^44^^,^^45^ however assessment of distress related to sexual complaints are quite rarely seen.^35^

Even though no significant gender differences were found for overall sexual satisfaction, some gender differences in sexual life after stroke are worth mentioning. The high prevalence of female sexual complaints confirm the need to highlight female sexuality after stroke, inadequately addressed in clinical practice and research.^12^^,^^16^^,^^23^ Women with sexual complaints were more satisfied with sexual life than men with sexual complaints, and men more often tended to experience sexual complaints as distressing. The results may indicate that these women, but not men, have found ways to cope with their sexual complaints. Moreover, adequate coping strategies may also explain that some patients were satisfied with their sexual life despite distressing complaints. This might be in agreement with Nilsson et al^46^ who found that some couples experienced positive changes in sexuality after stroke, explained as increased feelings of intimacy in the relationship. In contrast, some experienced good sexual function, but were nevertheless dissatisfied with sexual life, confirming that sexual satisfaction is more complex and goes beyond sexual function.^47^

The contribution of cognitive impairments to sexuality after stroke is unknown, although Korpelainen et al^48^ in a study of 50 stroke patients found cognitive deficits (assessment not given) not to be associated with sexual dysfunction. Despite this, one could assume that cognitive impairments can contribute to explain the low sexual satisfaction in the present cohort, and further investigation into this association is warranted.

### Strengths and Limitations

The main strength of this study is the inclusive, broad biopsychosocial and clinical approach when investigating sexual satisfaction after stroke in patients with cognitive impairments. Moreover, it explores sexuality in a younger cohort of stroke patients including women and singles who are underrepresented in earlier literature. Establishing a comfortable interview setting with a trained sexologist where patients could feel their sexual concerns were met professionally should be considered an advantage. Adaptation to participants' needs, using a person-centered approach, resulted in very few missing data and a high response rate.

A limitation is that the study is underpowered for conducting multivariable analysis for gender-subgroups; however, crude analyses provide information on gender-specific independent associations to sexual satisfaction. When exploring a broad range of independent variables in relation to satisfaction with sexual life, multiple testing can be an issue, and the results should be interpreted with this in mind.

### Implication for Clinical Practice and Research

A majority wanted follow-up for their sexual concerns, confirming the need to also address sexuality in cognitive stroke rehabilitation. A broad and multidisciplinary assessment and interventional approach integrating medical as well as psychosocial issues needs to be applied, and rehabilitation professionals and sexologists should not hesitate to raise this topic with stroke patients. It is recommended to include a generic screening addressing sexual life as standard routine to identify any need for further assessments. Personal and relational issues seem to be particularly important. The unmet sexual rehabilitation needs of stroke patients are likely partly explained by the lack of psychosocial support for emotional and relational issues related to sexuality. Therefore, evidence-based guidance for professionals in delivery of such support is needed. A knowledge gap is the absence of studies investigating associations between cognitive impairments and sexuality. Moreover, future research need to include partner perspective and dyadic patterns.

## CONCLUSION

The present study contributed with novel knowledge on aspects of sexuality and satisfaction in stroke patients in specialized cognitive rehabilitation. The wide range of variables associated with sexual satisfaction confirm the need for a broad biopsychosocial approach to sexuality poststroke. The contribution of intra- and interpersonal factors offer rehabilitation professionals as well as sexologists the opportunity to intervene.

## STATEMENT OF AUTHORSHIP

Conceptualization: J.K.V., H.S., M.I.N., J.K.S., A.Ø.G., K.S.F-M.; Methodology: J.K.V., M.I.N., J.K.S., A.Ø.G., K.S.F-M.; Formal Analysis: J.K.V., M.I.N., A.Ø.G.; Investigation: J.K.V., H.S.; Data curation: J.K.V., H.S.; Writing – Original Draft: J.K.V., H.S., K.S.F-M.; Writing – Review & Editing: J.K.V., H.S., M.I.N., J.K.S., A.Ø.G., K.S.F-M., Visualization: J.K.V., K.S.F-M.; Supervision: K.S.F-M.; Project Administration: J.K.V.; Funding Acquisition: J.K.V., J.K.S., K.S.F-M.
